# Normal spermatogenesis and fertility in *Spmip8* deficiency male mice

**DOI:** 10.1016/j.bbrep.2025.102406

**Published:** 2026-01-06

**Authors:** Zeling Zhang, Meihong Hu, Di Yan, Yuanqi Zhao, Wen Tao, Shengyuan Chen, Meijie Qi, Lei Luo, Xiaohua Jiang, Bo Xu, Shun Bai

**Affiliations:** Center for Reproduction and Genetics, Department of Obstetrics and Gynecology, The First Affiliated Hospital of USTC, Division of Life Sciences and Medicine, University of Science and Technology of China, Hefei, Anhui, 230001, China

**Keywords:** *Spmip8*, Male fertility, Spermatogenesis

## Abstract

Spmip8, also known as Tepp, is a protein-coding gene which highly conserved in the mammals. Although SPMIP8 has been reported to be highly expressed in the testis, the function of SPMIP8 in spermatogenesis and male fertility remain unknown. In this study, we used CRISPR/cas9-mediated genome editing system to generate *Spmip8*-deficient mice. The phenotype of *Spmip8* knockout (KO) male mice was performed by fertility tests, histology, and immunofluorescence. SPMIP8 is localization to the flagella of elongating spermatids in testis. *Spmip8* KO male mice exhibited normal fertility. No significant differences were found in sperm count, motility, morphology and kinematic parameters between WT and *Spmip8* KO mice. Furthermore, no detectable defects in spermatogenesis were found in KO mice. The transcription level of several *Spmip* genes (*Spmip1*, *Spmip2*, *Spmip3*, *Spmip7* and *Spmip11*) was elevated in the testes of *Spmip8* knockout mice, suggesting that *Spmip8* gene in male fertility could be compensated by other *Spmip* family members. Overall, the findings of this study suggest that *Spmip8* is not an essential gene for male fertility in mice. Our study helps researchers avoid duplication and repetitive work and explore genes that are integral to spermatogenesis and male fertility.

## Highlights

• *Spmip8* is a highly conserved gene in testes.
• *Spmip8*^*−/−*^ males showed normal testis volume, fertility, sperm count, and motility.
• *Spmip8*in male fertility could be compensated by other *Spmip* family members.

## 1 Introduction

Maintaining male fertility depends on the production of sperm capable of fertilization. In mammals, the spermatogenic process is complex and dynamic, including three sequential stages: the mitotic division of spermatogonia to produce spermatocytes, two rounds of meiosis to form spermatids, and the differentiation of the post-meiotic cells into mature sperm[[1], [2], [3]]. This process requires the coordinated expression of thousands of proteins within the testes, involving numerous testis-enriched proteins. Currently, researchers have identified approximately 2300 genes predominantly expressed in the testes[[4], [5], [6], [7]].However, the roles of these highly expressed protein-coding genes in spermatogenesis and male fertility remain unclear.

The sperm flagellum is a complex, microtubule-based structure called the axoneme, which is essential for motility. The stability and function of the axoneme's doublet microtubules (DMTs) are reinforced by a diverse array of microtubule inner proteins (MIPs)(Chen et al., 2023). Recent cryo-electron tomography studies have identified numerous sperm-specific MIPs (sperm-MIPs) in mammals, which are thought to provide the extra stability required for the powerful movements of sperm flagella[8]. *Spmip* gene family encodes several of these sperm-MIPs. While the functions of some family members have been linked to sperm motility and fertility, such as *Spmip7* and *Spmip10*, the roles of others remain poorly understood[9].

*Spmip8* refers to *Tepp*, is a protein-coding gene that exhibits evolutionary conservation in mammals, with a high degree of amino acid sequence similarity among human, mouse and rat[10]. The *Spmip8* gene encodes a 37 kDa protein that is specifically expressed in testis, prostate, and placenta. Additionally, SPMIP8, a component of the dynein-decorated doublet microtubules (DMTs) within the axoneme of the sperm flagellum, has the primary function of reinforcing and stabilizing the microtubule structure of the sperm flagellum[8]. This specific expression suggests that it may play a role in spermatogenesis and male fertility. However, the special function of SPMIP8 in the process of testicular development and spermatogenesis remains unclear. This study aims to investigate the specific role of *Spmip8* gene in the male reproductive process through a mouse model using CRISPR/Cas9 technology. However, our study shown *Spmip8* knockout mice were fertile and exhibited normal germ cell development, although SPMIP8 is an evolutionarily conserved and testis-specific protein. This suggests that *Spmip8* is not essential for male fertility in mice.

## 2 Method

### 2.1 Animals

Mice were housed at the Animal Center of the First Affiliated Hospital of the University of Science and Technology of China, with a maximum of five mice per cage. They were maintained under appropriate conditions including ventilation, light-dark cycle, and room temperature, with bedding changed regularly and cages cleaned. All experimental procedures described in this study were approved by the Animal Ethics and Welfare Committee of the First Affiliated Hospital of the University of Science and Technology of China.

### 2.2 Generation of *Spmip8* knockout mice

*Spmip8* knockout C57BL/6J mice were generated using the CRISPR/Cas9-mediated genome editing system by Cyagen Biosciences, Suzhou, China. Four sgRNAs were used in this study: gRNA-A1 (GGCCTAGGCAAATCCAGAGTTGG), gRNA-A2 (CCACATTCAGGATCTTGGTTAGG), gRNA-B1 (CATTCTAAATAGCTTTACTATGG), and gRNA-B2 (TAACCTGCCCACTTCCCATCAGG). Cas9 mRNA and sgRNAs were co-injected into the cytoplasm of mouse zygotes, resulting in *Spmip8* knockout mice. Genomic DNA to extracted to determine the pups’ genotypes. After obtaining heterozygous mice, F0 generation mice were housed together and continuously mated to produce homozygous gene-deficient mice.

### 2.3 Genotyping

Genotyping was conducted through PCR amplification following the clipping of the mouse toes (F1, 5′-GAGGGTCAGGAAGTAGAGTCAACA-3’; R1, 5′-TCCCACTGCTCAAGAGTACATACA-3’; F2, 5′-GAAAAGGCTGATTCTTCTATGGCT-3′). Following several generations of continuous breeding, homozygous mutant mice were eventually generated. The F0 generation mice were subjected to Sanger sequencing to verify the targeting and the results were analyzed using SnapGene (v.7.0.2).

### 2.4 Fertility test

To assess the fertility of knockout mice, 10-week-old *Spmip8*^−/−^ male mice were individually paired with two 10-week-old WT female mice over a period exceeding eight weeks. The number of offspring per litter was systematically monitored and recorded throughout this duration.

### 2.5 Detection of body and testes weight, sperm count, and sperm motility

Body and testes weights were measured using a high-precision electronic scale in 10-week-old *Spmip8*^−/−^ and WT male mice. For motility test, mature sperm from cauda epididymides were released in human tubal fluid (HTF) at 37 °C for 2 min and assessed using a computer-assisted sperm analysis (CASA) system (Hamilton Thorne Biosciences, USA). For count test, sperm samples from cauda epididymides were centrifuged and then resuspended in 4 % PFA for 10 min. Sperm were counted using a hemocytometer chamber under a light microscope.

### 2.6 Histological analyses

Testis and epididymis from 10-week-old *Spmip8*^−/−^ and WT mice were fixed in Bouin's solution at 4 °C for 24–48 h, and subsequently dehydrated through a graded ethanol series ranging from 70 % to 100 %. After clearing with xylene and embedded in paraffin, the specimens were sectioned at 5 μm and stained by hematoxylin and eosin (H&E; Sigma-Aldrich, USA). The samples were analyzed using a microscope (LEICA DM2500, Germany).

### 2.7 Quantitative RT-PCR

Total RNA was extracted from the testicular tissue of 10-week-old *Spmip8*^−/−^ and WT mice using TRIzol reagent (15596018, Thermo Fisher Scientific, MA, USA). Then, 1 microg of each sample reverse transcribed to cDNAs using All-In-One RT MasterMix (G492, ABM, Canada). The quantitative PCR was performed using the TransStart Green qPCR SuperMix (AQ131-01) on a LightCycler96 instrument (Roche, Swiss). The primers for qRT-PCR are listed as follow: *Spmip1* forward: 5′-GAGATGATGTTGCGCTACGAA-3′, reverse: 5′-ACGGGGTACATATCTGACAGAA-3’; *Spmip2* forward: 5′-GCGCATGATCGTCACAGGT-3′, reverse: 5′-ATGCTGGACGCTTTTCTCCTA-3’; *Spmip3* forward: 5′-GACCATCCGACTACGAGAATTTG-3′, reverse: 5′-TCCAGGGTAATAGCCTTTGATGT-3’; *Spmip4* forward: 5′-ATGGAGGTAATTCACGGCAGG-3′, reverse: 5′-TTGGCCGAATAGTTGTTTCGT-3’; *Spmip5* forward: 5′-CTCAAGCCAGCGGAGAGTAAA-3′, reverse: 5′-ACGCTTTCACACAGGGATTCA-3’; *Spmip6* forward: 5′-TGTCTCGGAACCGGACTCT-3′, reverse: 5′-GGAGCAAGGGCTTTCATATCC-3’; *Spmip7* forward: 5′-GCCAGCCAAACCAATCAATTT-3′, reverse: 5′-TTGTCCACATTGTCAGCGTCT-3’; *Spmip8* forward: 5′-GTGCCATCGAAGATTGGTCTAA-3′, reverse: 5′-CTCCGGCTTCAAGTACCGC-3’; *Spmip9* forward: 5′-TACCAAAGCTCATACATGGTGGA-3′, reverse: 5′-CTTCTGAAAGCAGGATAGCCTTC-3’; *Spmip10* forward: 5′-GATGACGGTCCGGCCTTAC-3′, reverse: 5′-GCAAGTGAATATCGTCCCACC-3’; *Spmip11* forward: 5′-CAAGGCTTCCCCCGATTATCT-3′, reverse: 5′-ACGTTTTTAACAACACCTTTCGC-3’; *β-Actin* forward: 5′-CCAGAGCAAGCGAGGTATCC-3′, reverse: 5′-GCCACACGCAGCTCATTGTA-3’.

### 2.8 Western blot analysis

Testicular tissues from 10-week-old wild-type and *Spmip8*^−/−^ mice were lysed on ice using RIPA buffer supplemented with a protease inhibitor cocktail. After centrifugation to remove debris, protein concentration was determined by BCA assay. Equal amounts of protein (30 μg) were separated by 10 % SDS-PAGE and transferred to a PVDF membrane. The membrane was blocked with 5 % nonfat milk in TBST for 1 h, then incubated overnight at 4 °C with primary antibodies against SPMIP8 (1:1000, HPA062092, Sigma, Germany) and α-TUBULIN (1:5000, 11224-1-AP, Proteintech, China). After washing, the membrane was incubated with HRP-conjugated secondary antibodies for 1 h at room temperature. Protein bands were visualized using an enhanced chemiluminescence (ECL) system.

### 2.9 Immunofluorescence

Testes from 10-week-old *Spmip8*^−/−^ and WT mice were fixed in 4 % Paraformaldehyde (PFA), dehydrated, and embedded in paraffin. After deparaffinization and rehydration, antigen retrieval was performed in citrate buffer. The sections were blocked with 10 % BSA and incubated with primary antibodies overnight at 4 °C. Primary antibodies used were: anti-SPMIP8 (1:200, HPA062092, Sigma, Germany), anti-γ-H2AX (1:100, 16–202A, Merck Millipore, USA), and PNA-lectin (1:5000, RL-1072, Vector Labs, USA). After washing with PBS, sections were incubated with corresponding fluorescently-conjugated secondary antibodies for 2h at 37 °C. Slides were mounted with mounting medium containing DAPI (F6057, Sigma, USA) and observed under a fluorescence microscope (Leica DM300, Wetzlar, Germany).

### 2.10 Phylogeny analysis

For the phylogenetic tree analysis, amino acid sequences from multiple species were obtained from the NCBI database, aligned by the ClustalW algorithm, and constructed by the neighbor-joining method with 1000 bootstrap replicates using MEGA X software.

### 2.11 Statistical analysis

All data were reported as mean values with standard deviations (SD). Statistical analysis was performed using GraphPad Prism 9, with a p-value <0.05 considered statistically significant.

## 3 Result

### 3.1 SPMIP8 is expressed specifically in testis

Phylogeny analysis identified that SPMIP8 is highly conserved among mammals (Fig. 1A). To investigate the SPMIP8 expression pattern, we analyzed previous transcriptomics data from different mouse tissues and spermatogenic cells (Fig. 1B–D). Single-cell RNA-seq data showed that *Spmip8* was high expressed in early and late spermatids in humans [11](Fig. 1B). In line with human data, *Spmip8* is specifically expressed in the mouse testis, with expression elevated at postnatal day (P) 21 and peaking in spermatids, indicating that it may play a special role in spermiogenesis [12,13];(Fig. 1C–E).

**Fig. 1:**
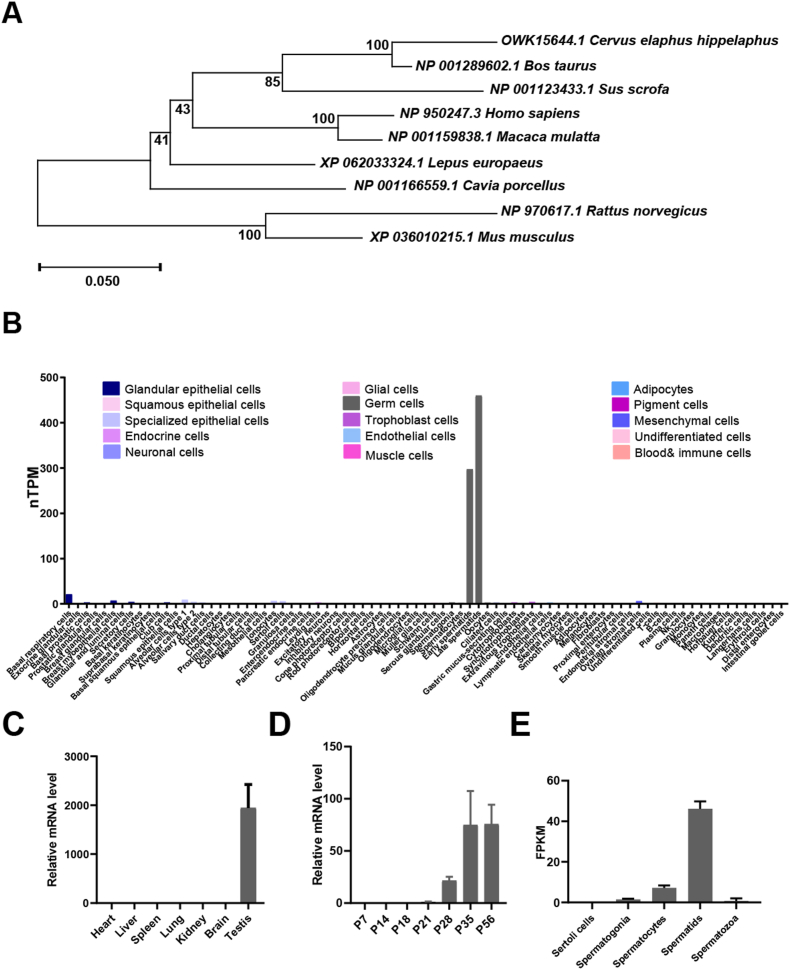
*Spmip8* is a conserved gene that is highly expressed in spermatids. (A) Phylogenetic trees of SPMIP8 homologous proteins in mammalian species. The numbers in the dendrogram are bootstrap values (%). (B) Expression pattern of *Spmip8* mRNA from published scRNA-seq in humans. (C) Expression pattern of *Spmip8* mRNA from published mouse RNA-seq data of various types of mouse tissues. (D) QRT-PCR analyses of *Spmip8* mRNA levels in developing testes at postnatal Day 7 (P7), P14, P18, P21, P28, P35 and P56, n = 3. (E) Expression *Spmip8* mRNA data of isolated spermatogenetic cells from published mouse RNA-seq data.

### 3.2 Generation and validation of *Spmip8* knockout mice

By CRISPR/Cas9 genome editing technology, we generated a *Spmip8* mutation mice carrying 8988bp deletion between exon 2 to 8 (Fig. 2A). Sanger sequencing confirmed the successful knockout in *Spmip8* gene (Fig. 2B). After genotyping, heterozygous *Spmip8* knockout (hereafter referred to as *Spmip8*^*−/−*^) mice underwent mating to generate homozygous mutant mice (Fig. 2C). Deletion of *Spmip8* in *Spmip8*^*−/−*^ mice was confirmed by qRT-PCR analyses (Fig. 2D). Western blot analysis of testis confirmed the complete absence of the SPMIP8 protein in *Spmip8*^*−/−*^ mice (Fig. 2E). Immunostaining of adult testis revealed that SPMIP8 was present in the flagellar of elongating spermatids, while no signal was detected in the flagellar of *Spmip8*^*−/−*^ spermatids (Fig. 2F).

**Fig. 2:**
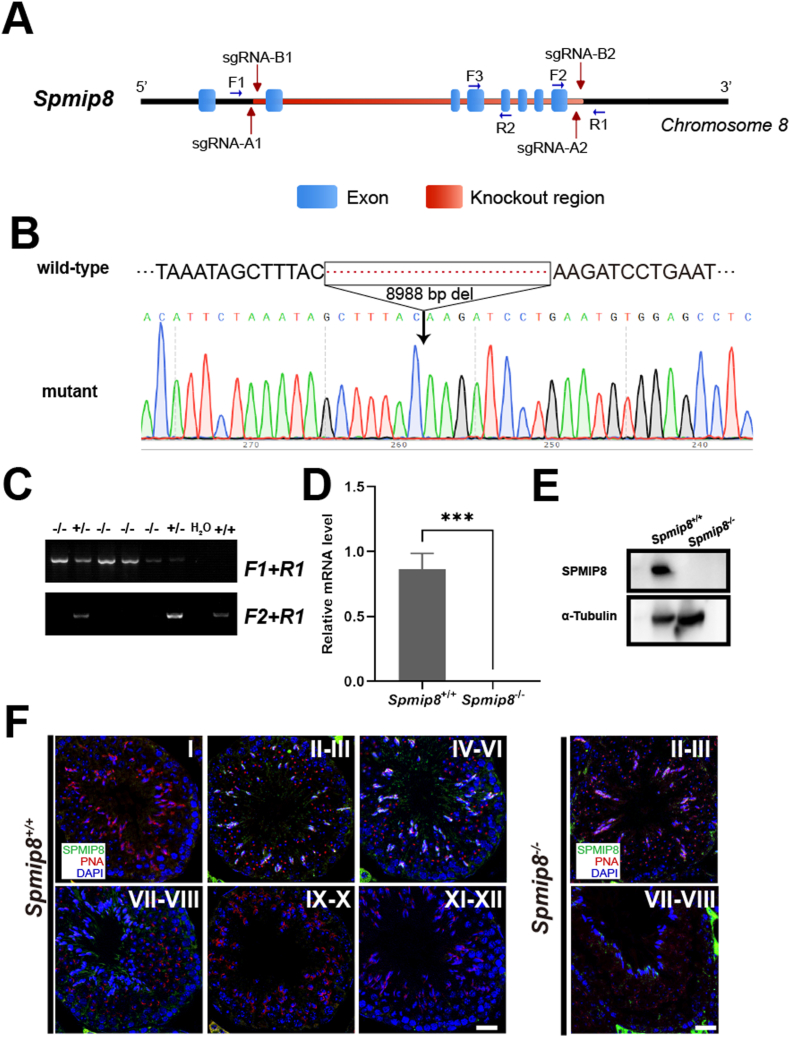
Generation and validation of *Spmip8* knockout mice. (A) Schematic diagram of *Spmip8*^−/−^ mouse creation. (B) Sanger sequencing of genomic DNA shows a deletion in the *Spmip8*^-^ gene. (C) *Spmip8*^−/−^ mice were identified by genomic PCR. (D) *Spmip8*^-^ transcripts were not detected in adult *Spmip8*^−/−^ testes, n = 3 for each genotype. (E) Western blot analysis the SPMIP8 protein in *Spmip8* knockout mice. α-TUBULIN was used as a loading control. (F) Immunofluorescence staining of SPMIP8 (green), PNA (acrosome, red) in testis sections from 10-week-old WT and *Spmip8*^−/−^ mice. Magnification ×40 in the panels. DAPI (blue) stains the nuclei. The head signal in elongating spermatids is non-specific, as it appears in both WT and *Spmip8*^−/−^ testis sections. Scale bar: 50 μm ∗∗∗*P* < 0.001.

### 3.3 *Spmip8* knockout mice showed normal spermatogenesis and male fertility

*Spmip8* knockout males showed no significant abnormalities in growth, development, or behavior. No significant differences in the testis weight (Fig. 3A and B), body weight (Fig. 3C) and testis/body weight ratio (Fig. 3D) between *Spmip8*^−/−^ and WT male mice. To assess whether the *Spmip8* deficiency affects male fertility, we carried out mating tests and observed there was no significant difference in litter sizes between *Spmip8*^−/−^ males and WT males (Fig. 3E). To further determine the spermatogenesis defects in *Spmip8*^−/−^ males, we performed H&E staining of testis and epididymis (Fig. 3F; Fig. 4A). The results showed that the epididymides of the *Spmip8*^−/−^ males contained a normal sperm production and exhibited typical seminiferous tubules with all types of germ cells, from spermatogonia to spermatozoa. To determine whether spermatogenesis is affected, we co-immunostained γ-H2AX with DAPI on testicular sections and staged seminiferous tubules. All stages of spermatogenic cells were observed in testis sections from 10-week-old WT mice and *Spmip8*^−/−^ mice (Fig. 3G).

**Fig. 3:**
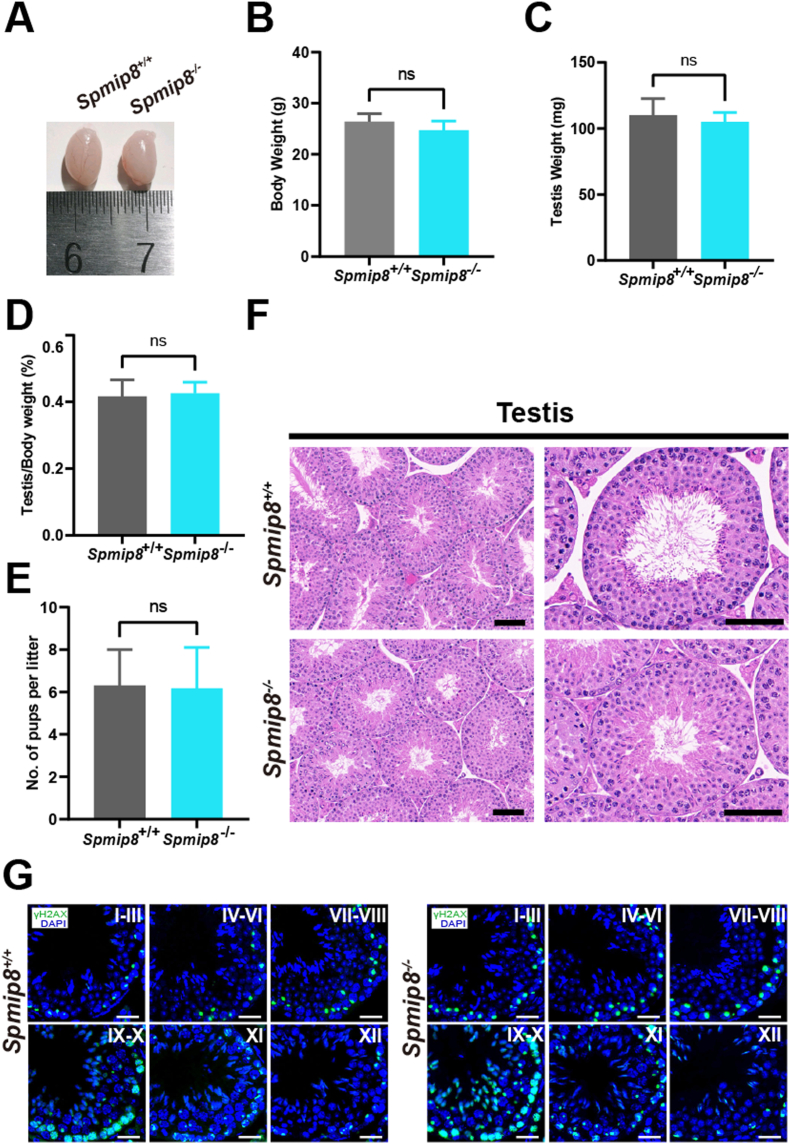
*Spmip8*^−/−^ mice show normal spermatogenesis. (A) Representative image of WT and *Spmip8*^−/−^ testes from 10-week-old mice. (B) Body weight of 10-week-old WT and *Spmip8*^−/−^ mice. n = 5 for each genotype. (C) Testis weight from 10-week-old WT and *Spmip8*^−/−^ mice. n = 5 for each genotype. (D) The ratio of testis weight to body weight from 10-week-old WT and *Spmip8*^−/−^ mice. n = 5 for each genotype. (E) Number of pups per litter from WT and *Spmip8*^−/−^ males. (F) H&E staining of testes and epididymides from 10-week-old WT and *Spmip8*^−/−^ mice. Magnification ×20 in the left panels and ×40 in the right panels. Scale bar: 50 μm. (G) Immunofluorescence staining for γ-H2AX (green) in testis sections from 10-week-old WT and *Spmip8*^−/−^ mice. DAPI (blue) stains the nuclei. Magnification ×40 in the panels. Scale bar: 50 μm. NS, not significant.

**Fig. 4:**
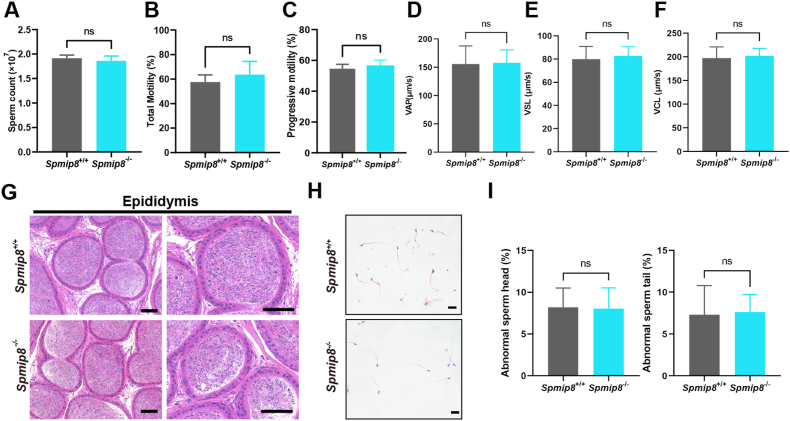
*Spmip8* deficiency does not affect sperm quality. (A) Sperm count from 10-week-old WT and *Spmip8*^−/−^ male mice. n = 4 for each genotype. (B) Sperm total motility from 10-week-old WT and *Spmip8*^−/−^ male mice. n = 5 for each genotype. (C) Sperm progressive motility from 10-week-old WT and *Spmip8*^−/−^ male mice. n = 5 for each genotype. (D) Average path velocity (VAP) of sperm from 10-week-old WT and *Spmip8*^−/−^ male mice. n = 4 for each genotype. (E) Straight line velocity (VSL) of sperm from 10-week-old WT and *Spmip8*^−/−^ male mice. n = 4 for each genotype. (F) Curvilinear velocity (VCL) of sperm from 10-week-old WT and *Spmip8*^−/−^ male mice. n = 4 for each genotype. (G) H&E staining of epididymides from 10-week-old WT and *Spmip8*^−/−^ mice. Scale bar: 50 μm. (H) H&E staining shows sperm morphology from 10-week-old WT and *Spmip8*^−/−^ male mice. Scale bar: 10 μm. NS, not significant.

### 3.4 *Spmip8* deficiency does not affect sperm quality

Analyses of sperm counts and motility did not show any significant difference between *Spmip8*^−/−^ and WT mice (Fig. 4B and C). To evaluate whether sperm morphology was changed in *Spmip8*^−/−^ mice, we performed HE staining of mature sperm head and flagella. The results showed that no significant abnormalities in sperm morphology were detected in *Spmip8*^−/−^ mice (Fig. 4D). No genotype-dependent differences were detected in any sperm motility or kinematic parameters, including total motility, progressive motility, average path velocity (VAP), straight line velocity (VSL), or curvilinear velocity (VCL). Furthermore, no elevation in the percentage of head or tail abnormalities in *Spmip8*^−/−^ sperm relative to WT (Fig. 4E).

### 3.5 Functional redundancy of *Spmip genes*

To further investigate why *Spmip8* is not essential to male fertility, we performed the mRNA expression of *Spmip* family genes in *Spmip8*^−/−^ males. The expression of *Spmip1*, *Spmip2*, *Spmip3*, *Spmip7* and *Spmip11* was upregulated in the testes of *Spmip8*^−/−^ mice compared with that in the testes of WT mice (Fig. 5).

**Fig. 5:**
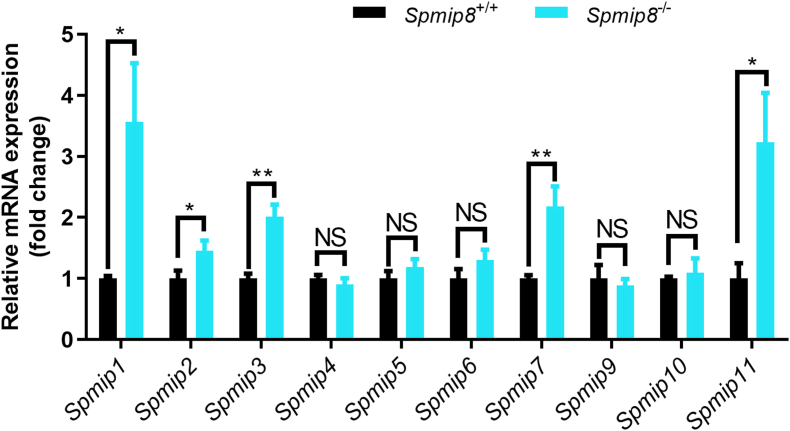
Functional compensation of *Spmips*. QRT-PCR showing the mRNA expression levels of 10 *Spmip* genes in testes from 10-week-old WT and *Spmip8*^−/−^ mice. n = 3 for each genotype. NS, not significant. ∗*P* < 0.05. ∗∗*P* < 0.01.

## 4 Discussion

In this study, we generated *Spmip8* mutant mice and investigated the function of SPMIP8 in spermatogenesis and male fertility. Although SPMIP8 is specifically expressed in the testes, we did not observe significant fertility impairment in *Spmip8* knockout mice. Histological analysis showed normal germ cell development in the testes and epididymides of *Spmip8* mutant mice. Additionally, *Spmip8* knockout mice exhibited relatively normal sperm parameters. These data indicate that *Spmip8* gene is dispensable for mouse spermatogenesis and male fertility.

Recently, Leung identified over 60 proteins decorating sperm DMTs, including 14 SPMIP proteins. Among these SPMIP proteins, 11 of which (SPMIP1, SPMIP2, SPMIP3, SPMIP4, SPMIP5, SPMIP6, SPMIP7, SPMIP8, SPMIP9, SPMIP10, SPMIP11) were observed in bovine sperm, and 3 SPMIPs (SPMIP11L, SPMIP12, SPMIP13) that are specific to sea urchin sperm[8]. It is worth noting that *Spmips* were mainly expressed in spermatids in mammals [11,13]. Importantly, SPMIP7 is believed to be linked to male infertility and a target for novel male contraceptives[9]. In addition to SPMIP7, knockout of SPMIP10 did not reduce overall fertility but affect sperm motility[8]. Other sperm MIPs, such as SPMIP6 and SPMIP9 were not required for male fertility in Ref. [14]. Although recent study has shown TEPP (SPMIP8) deficiency was dispensable for male reproduction[15], whether it affect the function of spermatogenesis remains unknown. In this study, we performed histological analysis to detect different germ cell development in *Spmip8* knockout mice and found there were no defects in Sertoli cells, spermatogonia, meiosis, and post-meiosis cells. Additionally, *Spmip8* knockout mice did not show abnormalities in sperm count and morphology, indicating SPMIP8 is not indispensable for spermatogenesis. Furthermore, the transcription level of several *Spmip* genes (e.g., *Spmip1*, *Spmip2*, *Spmip3*, *Spmip7* and *Spmip11*) was elevated in the testes of *Spmip8* knockout mice, suggesting that *Spmip8* gene in male fertility could be compensated by other *Spmip* family members.

In conclusion, we generated highly conserved and testis-expressed *Spmip8* knockout mice and demonstrated that SPMIP8 is not essential for spermatogenesis and male reproductive capability in mice. Our results could help researchers to avoid research duplication and focus on genes indispensable for testis development and male fertility.

## Author contributions

ZLZ, MHH, BX and SB designed the research study. ZLZ, MHH, YQZ, MJQ, WT and SB contributed to the data acquisition. ZLZ, DY, LL, SYC, XHJ, BX and SB analyzed the data. ZLZ and SB wrote the paper. All authors approved the final manuscript.

## Declaration of competing interest

The authors declare that they have no known competing financial interests or personal relationships that could have appeared to influence the work reported in this paper.

## Figures and Tables

**figs1:**
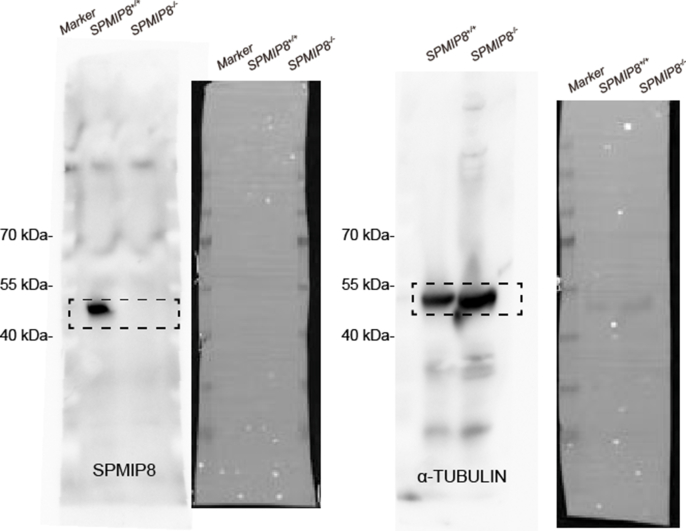


**figs2:**
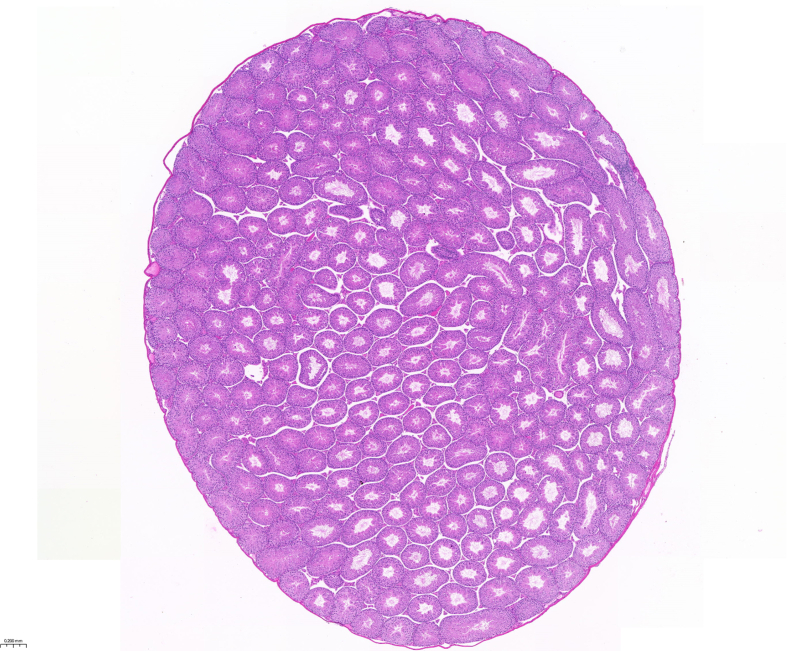


**figs3:**
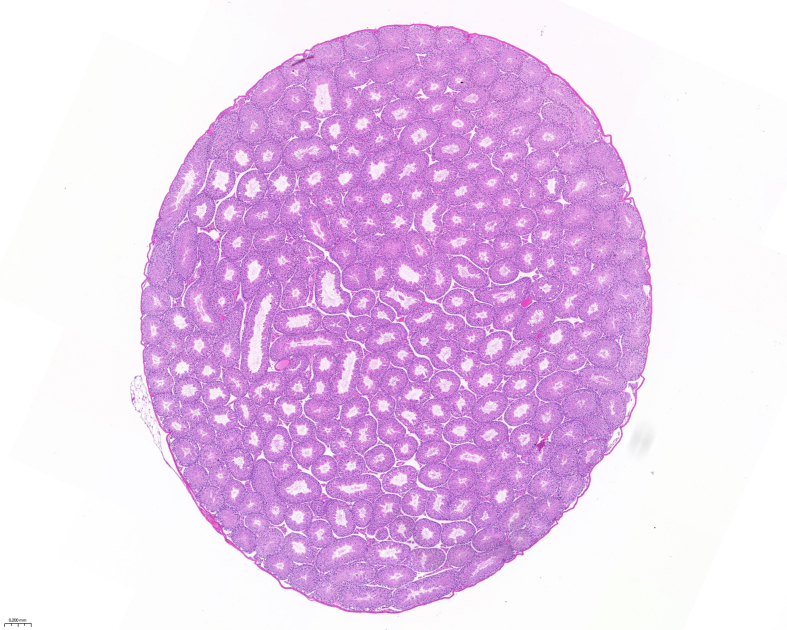


**figs4:**
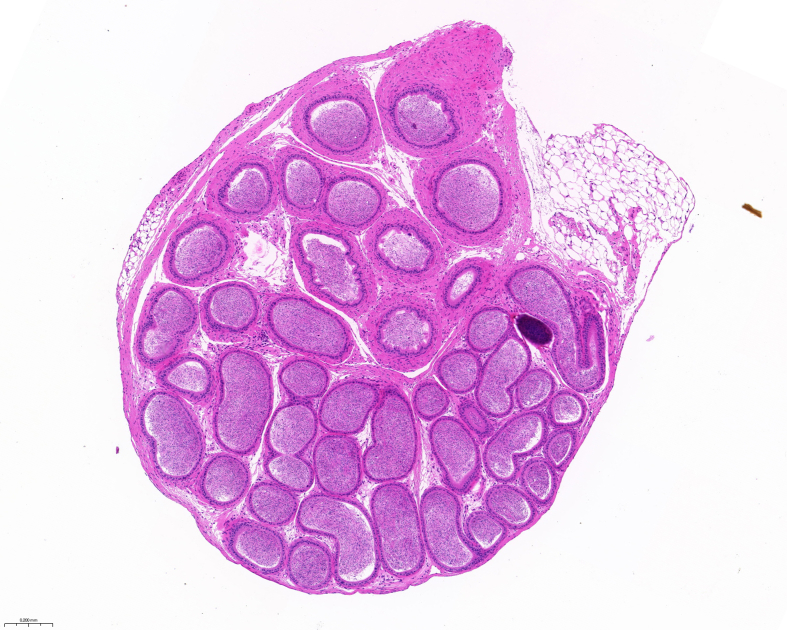


**figs5:**
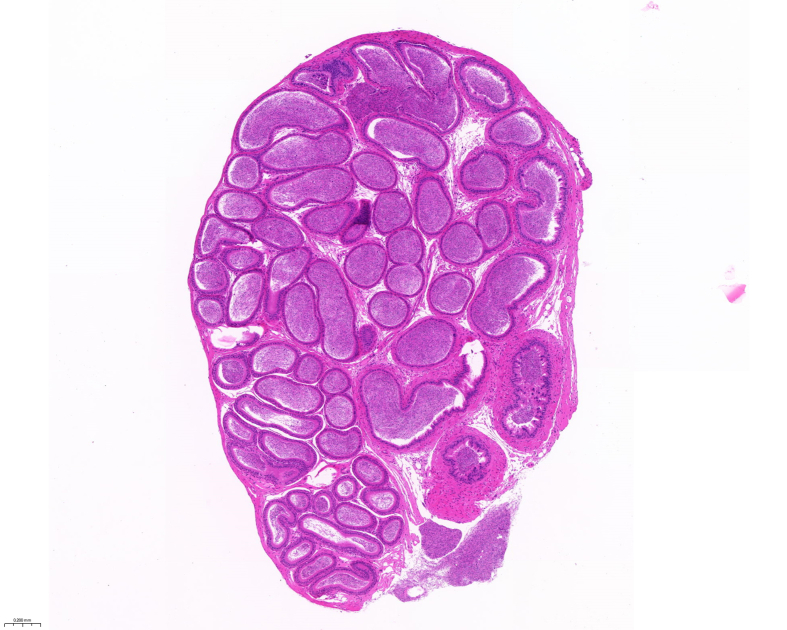


## Data Availability

Data will be made available on request.
