# Supplementary material for: Normal spermatogenesis and fertility in Spmip8 deficiency male mice
Source: Biochem Biophys Rep. 2026 Jan 6;45:102406. doi: 10.1016/j.bbrep.2025.102406 (PMC12808527; doi:10.1016/j.bbrep.2025.102406)

Figure 3F

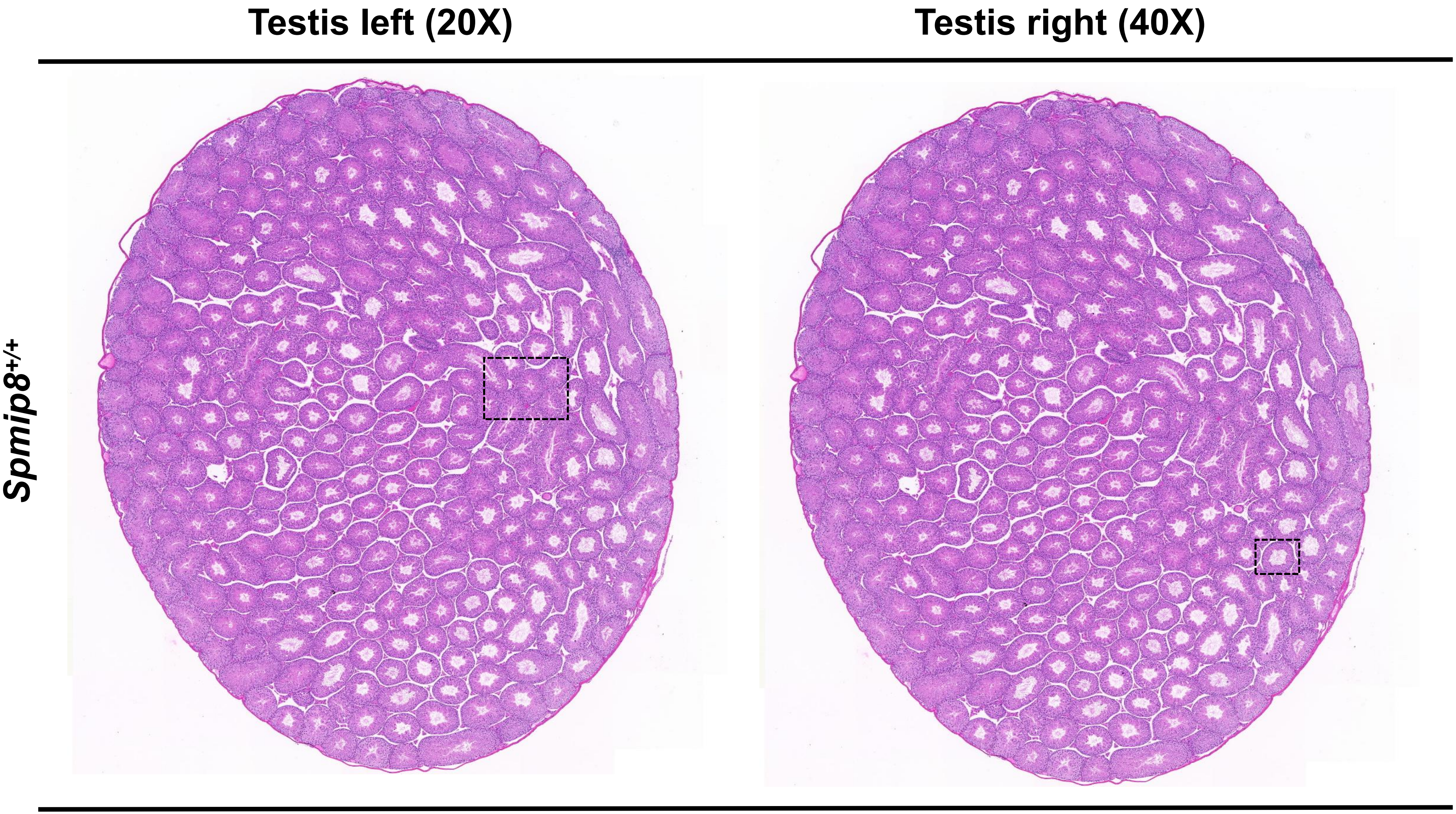

Figure 3F

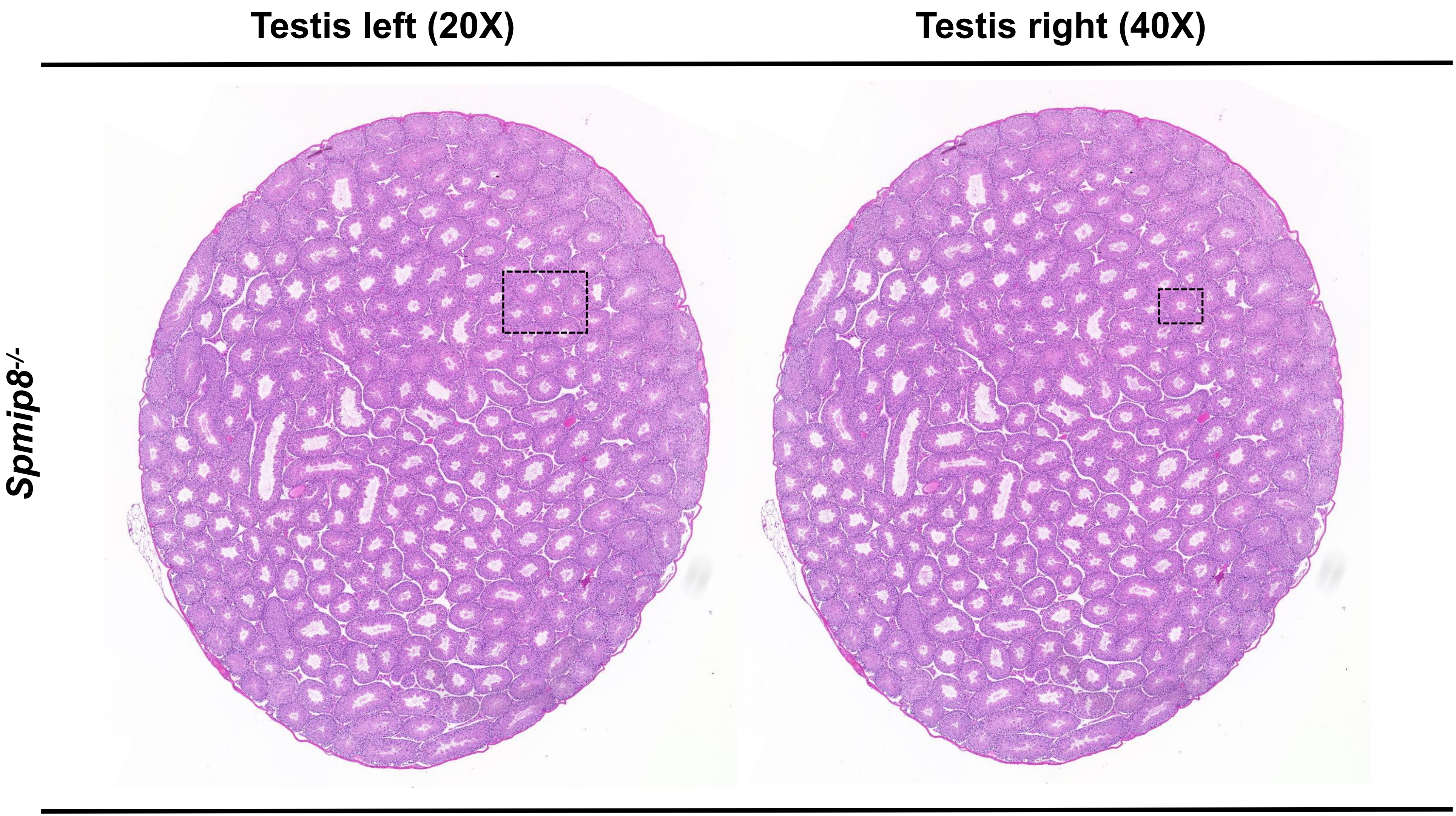

Figure 4G

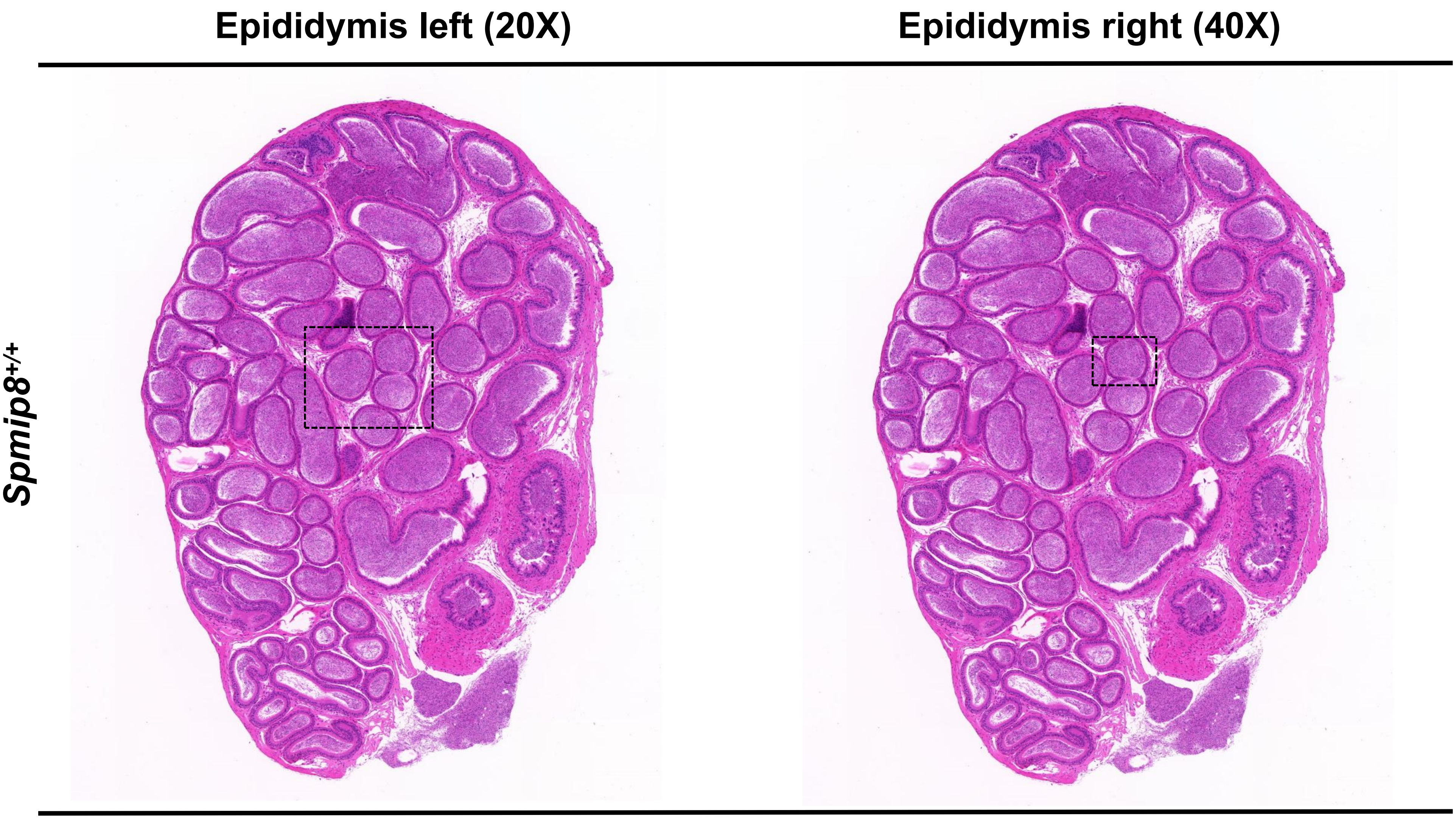

Figure 4G

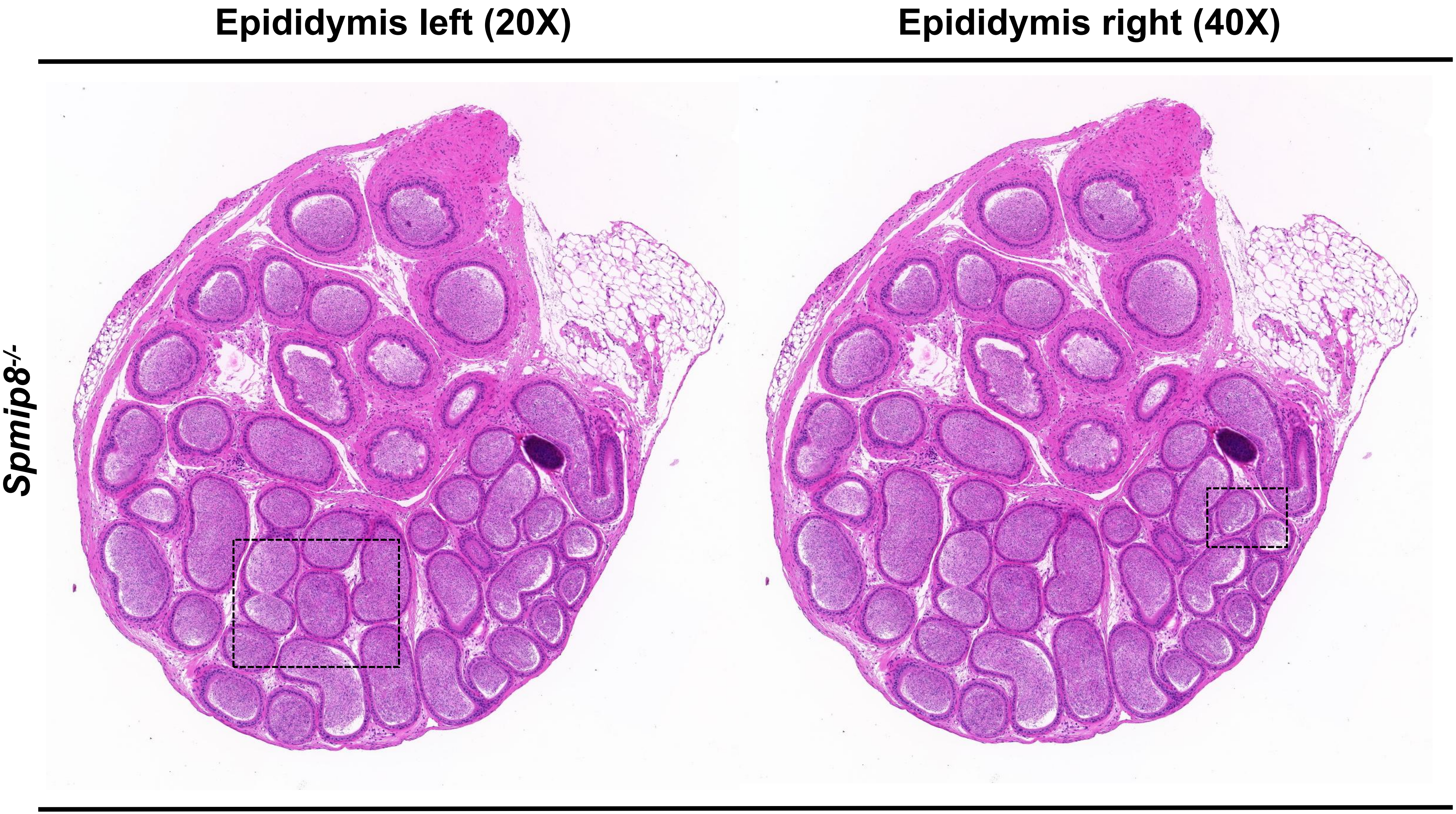

Supplement: Multimedia component 1 [file mmc1.pdf]
